# Seasonal dynamics in picocyanobacterial abundance and clade composition at coastal and offshore stations in the Baltic Sea

**DOI:** 10.1038/s41598-022-18454-8

**Published:** 2022-08-22

**Authors:** Javier Alegria Zufia, Catherine Legrand, Hanna Farnelid

**Affiliations:** 1 Department of Biology and Environmental Science, Centre for Ecology and Evolution in Microbial Model Systems (EEMiS), Linnaeus University, Kalmar, Sweden; 2 School of Business, Innovation and Sustainability, Halmstad University, Halmstad, Sweden

**Keywords:** Biodiversity, Ecological genetics, Microbial ecology, Ecology, Environmental sciences

## Abstract

Picocyanobacteria (< 2 µm in diameter) are significant contributors to total phytoplankton biomass. Due to the high diversity within this group, their seasonal dynamics and relationship with environmental parameters, especially in brackish waters, are largely unknown. In this study, the abundance and community composition of phycoerythrin rich picocyanobacteria (PE-SYN) and phycocyanin rich picocyanobacteria (PC-SYN) were monitored at a coastal (K-station) and at an offshore station (LMO; ~ 10 km from land) in the Baltic Sea over three years (2018–2020). Cell abundances of picocyanobacteria correlated positively to temperature and negatively to nitrate (NO_3_) concentration. While PE-SYN abundance correlated to the presence of nitrogen fixers, PC-SYN abundance was linked to stratification/shallow waters. The picocyanobacterial targeted amplicon sequencing revealed an unprecedented diversity of 2169 picocyanobacterial amplicons sequence variants (ASVs). A unique assemblage of distinct picocyanobacterial clades across seasons was identified. Clade A/B dominated the picocyanobacterial community, except during summer when low NO_3,_ high phosphate (PO_4_) concentrations and warm temperatures promoted S5.2 dominance. This study, providing multiyear data, links picocyanobacterial populations to environmental parameters. The difference in the response of the two functional groups and clades underscore the need for further high-resolution studies to understand their role in the ecosystem.

## Introduction

Unicellular picocyanobacteria (< 2 µm in diameter) belonging to the *Synechococcus*/*Cyanobium/Synechocystis* genus (SYN) are highly adaptable and widespread in aquatic ecosystems^1^. The genus is polyphyletic^2,3^ and is characterized by a high diversity in terms of pigment content^4^, taxonomy^5^ and physiology^6^. SYN populations are a major contributor to carbon flux in oligotrophic oceans^7,8^ as well as coastal, estuarine and freshwaters^9–11^. However, there are relatively few studies that focus on SYN dynamics and community composition in freshwater^12–14^ and estuaries^15–17^ despite showcasing higher diversity than their marine counterparts ^14^. Under climate change scenarios, the abundance of SYN cells is expected to increase^1,18^ while the rest of the phytoplankton community biomass is expected to decrease^19,20^. Understanding the distribution and dynamics of SYN ecotypes is therefore of high interest for evaluating ecosystem consequences.

Distribution and seasonal occurrence of marine phylogenetic clades have been linked to environmental factors, increasing our understanding on marine SYN dynamics and distribution^21–23^. SYN populations are composed of unicellular phycoerythrin (PE)-rich cells (PE-SYN, adapted to blue and green light) and phycocyanin (PC)-rich cells (PC-SYN, adapted to red light). It has been observed that PE-SYN are more prevalent in low turbidity waters (generally open waters), while PC-SYN are better adapted to turbid waters (generally coastal waters)^24–28^. Other factors, such as water column stability and stratification can also have a significant effect on the PE:PC ratios^11,29^. Peak SYN cell abundances are normally recorded during periods of warm temperatures and low nutrient concentrations^8,30^. SYN cells may use ammonium (NH_4_) from nitrogen (N_2_)-fixers or from regeneration through ammonification as main source of nitrogen^31–34^ or can take up dissolved organic nitrogen (DON) directly^35–37^. Other nutrients such as phosphate (PO_4_) have also been observed to have an effect on picocyanobacterial dynamics and distribution^38^.

Pigment related genes *cpcBA*, *mpeBA*, and *mpeW* have been used to describe SYN diversity^39^. Studies based on 16S rRNA, 16S-23S internally transcribed spacer (ITS), *petB*, *psbB*, *rpoC1*, narB genes as well as full genome analysis have provided more detailed phylogeny despite not being directly related to the pigment content or other morphological characteristics^40–47^. Phylogenetically, SYN populations can be divided into three subclusters: S5.1, composed strictly of marine strains, S5.2, containing both marine and brackish adapted strains and S5.3 containing mainly oceanic strains from surface waters^21,48–51^. These subclusters can be further divided into 20 well defined clades, which are closely related to the physio-ecological characteristics of SYN^52,53^. In addition, there are a number of brackish and freshwater clades that are not taxonomically assigned to known subclusters^12,14^.

The Baltic Sea is characterized by a salinity gradient from 2.9 PSU in the Bothnian Bay to 22 PSU in the Kattegat. In the Baltic Proper, picocyanobacterial blooms take place during the summer at temperatures up to 20–24 °C and nitrogen limited conditions^54–56^. Peak abundances during this period reach up to 10^5^ cells mL^−1^^55,57^ corresponding to 21% contribution to the total phytoplankton community in terms of Chl *a*^58^ and 56% in terms of carbon biomass^52^. The diversity of the picocyanobacterial community in the Baltic Sea has been characterized by amplification and sequencing of the V4-V5 16S rRNA gene hypervariable region (e.g.^6,15,17,59^). SYN sequences can dominate the sequence libraries^6,17^. During summer, the SYN populations are dominated by brackish strains from the S5.2 in the North, and there is a transition towards a community dominated by S5.1 at 13–16 PSU in the South^17^. Amplification and sequencing of the pigment genes for PE (*cpeBA*) and PC (*cpcBA*) showed that the Baltic Sea is equally populated by PE-SYN and PC-SYN in the upper 30 m of the water column^29^. In the summer, the SYN population is dominatied by an ecotype with a novel pigment gene which was recently characterized by a metagenome-assembled genome (MAG) reconstructed from the Baltic Proper (BACL30)^16^. The discovery of this unique brackish strain is linked to the existence of a global brackish microbiome^60,61^. The current data suggests that the high physiological diversity of the picocyanobacterial community shows adaptation to different temperatures and nutrient regimes^6^ but few annual studies have been conducted which limits the understanding of seasonal SYN dynamics.

Recently, Huber et al.^62^ showed that the hypervariable region V5-V7 of the 16S rRNA gene provided high resolution of picocyanobacterial diversity in both marine and freshwater environments. An accurate phylogenetic clade classification over multiple years in the dynamic Baltic Sea can help to disentangle the drivers of SYN and fill knowledge gaps of brackish and freshwater strains. Here we monitored the abundance of SYN cells with flow cytometry and studied its clade community composition at high-resolution during three consecutive years at a coastal and an offshore station. The goal was to study the relationship between SYN pigment ecotypes and phylogenetic clades with environmental parameters in a brackish environment.

## Results

### Environmental conditions

**Figure 1:**
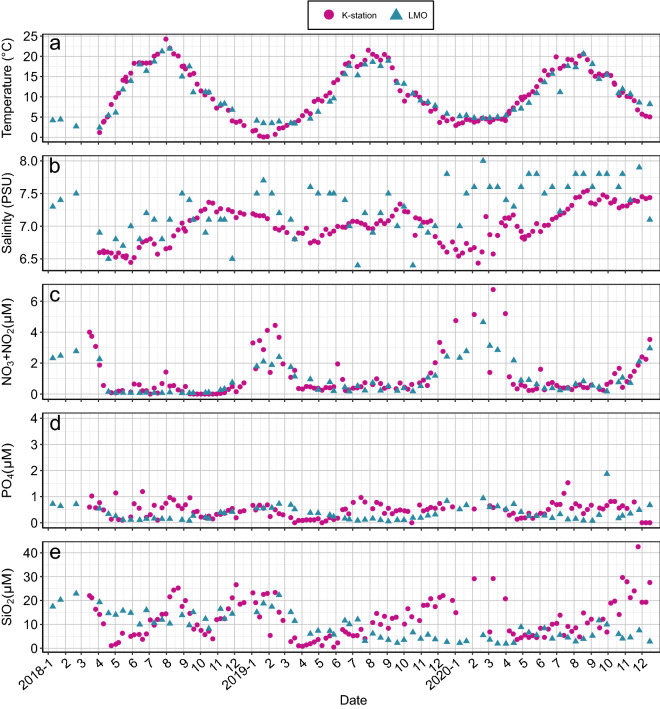
K-station circles and LMO (triangles) measurements for (**a**) temperature (°C), (**b**) salinity (PSU), (**c**) NO_3_ (µM), (**d**) PO_4_ (µM) and (**e**) SiO_2 _(µM)

Sampling was carried out every second week at the Linnaeus Microbial Observatory (LMO), an offshore station and weekly at the K-station, a coastal station during 2018–2020. Seawater temperature ranged from 0 to 5 °C during winter at both stations and increased during spring (~ 3–15 °C) and summer (~ 15–20 °C; Fig. 1A). In 2018, there was an earlier increase of temperature during summer compared to 2019 and 2020 (average 3 °C higher in the beginning of June) and the maximum temperatures during the study period were 24 and 22 °C at the K-station and LMO respectively. At LMO, the increase of temperature during summer resulted in stratification (mixed layer approx. depth: 20 m) that lasted until early to mid-autumn (stratification index (N^2^): 0.2–0.66 × 10^–3^ s^−2^; Supplementary Fig. S2A and B). In 2018, early signs of stratification were observed during spring due to the exceptionally high temperatures. Salinity ranged between 6.5 and 8 PSU (Fig. 1B). At the K-station, salinity increased during the spring to summer period while no clear seasonality was observed at the LMO. Inorganic nitrogen (NO_3_) concentrations ranged between < 0.06 (detection limit) and 5 µM, with the highest concentrations observed at the K-station during winter, which sharply declined upon temperature increase during spring (Fig. 1C). Phosphate concentrations (PO_4_) decreased during spring (from 1 to 0.2 µM) and remained low throughout the summer with occasional peaks up to 1 µM at the K-station (Fig. 1D). At the K-station, silicate (SiO_2_) showed a strong seasonality ranging from 4 to 25 µM with peaks in summer and early autumn (Fig. 1E). Silicate did not show a clear seasonality at LMO. Concentrations ranged between 10 and 20 µM during 2018, sharply declined during spring in 2019 and remained stable around 5 µM throughout 2020.

### Phytoplankton dynamics

**Figure 2:**
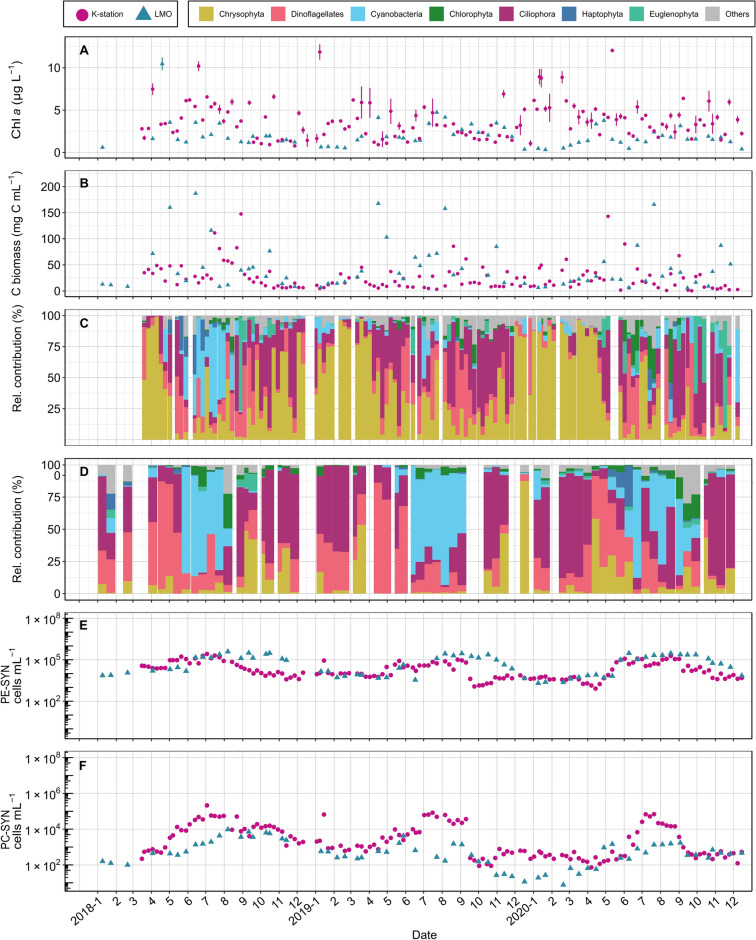
K-station and LMO measurements for (**a**) Chl *a* (µg L^−1^), (**b**) total phytoplankton (> 5 µm in diameter) carbon biomass concentration (mg C mL^−1^) based on microscopy, (**c**) K-station and (**d**) LMO relative contribution of phytoplankton divisions (> 5 µm in diameter) based on microscopy, (**e**) PE-SYN (cells mL^−1^) and (**f**) PC-SYN (cells mL^−1^).

Chlorophyll *a* (Chl *a*) concentration was generally higher at the K-station (1–7 µg L^-1^) compared to LMO (1–4.5 µg L^−1^; Fig. 2A). At the K-station, phytoplankton carbon biomass increased from 0.4 mg C mL^−1^ in the autumn to winter period up to 50.0 mg C mL^−1^ in the spring and 147.7 mg C mL^−1^ in the summer (Fig. 2B). The phytoplankton community was dominated by diatoms during autumn, winter and first half of the spring (Fig. 2C). During the second half of the spring the community transitioned to ciliates and dinoflagellates dominance. N_2_-fixers (filamentous cyanobacteria) were only observed during the summer 2018, reaching 111.3 mg C mL^−1^ in July. At the LMO, the maximum carbon biomass increased from 5.9 mg C mL^−1^ in the autumn and winter up to 597.4 mg C mL^−1^ in the spring and 167.7 mg C mL^−1^ in the summer (Fig. 2B). During the autumn, winter and spring, the community was dominated by ciliates and dinoflagellates (Fig. 2D), and occasionally by diatoms. During the summer the phytoplankton community was dominated by N_2_-fixers, reaching 111.3 mg C mL^−1^ total carbon biomass.

### Picocyanobacterial dynamics

PE-SYN concentration was low in the winter (LMO: 2.7 × 10^2^ cells mL^−1^, K-station: 8.2 × 10^2^ cells mL^−1^) and had peak abundances in the period from spring to summer (up to 3.8 × 10^5^ cells mL^−1^) and summer to autumn (up to 2.6 × 10^5^ cells mL^−1^) at the K-station and LMO respectively (Fig. 2E). PC-SYN increased from low concentrations in the winter (K-station: 72 cells mL^−1^, LMO: 8 cells mL^−1^) to peak concentrations during spring and summer (K-station: 2.1 × 10^5^ cells mL^−1^, LMO: 4.8 × 10^3^ cells mL^−1^; Fig. 2F).

To analyze the effect of each independent variable on PE-SYN and PC-SYN abundance, we used the partial least square regression (PLS) method to establish a regression equation. The number of components utilized for each equation was 2 and 6 for PE-SYN and PC-SYN respectively. Only the independent variables with variable importance in projection (VIP) > 1 were considered relevant for the final equation (Table 1). The model for PE-SYN explained 38% of the cell abundance seasonal changes with 52% variation of the independent variables. The regression equation between PE-SYN cell abundance and the independent variables was (1):

1 $$y = - 0.05 \cdot {\text{NO}}_{3} \left( {\mu {\text{M}}} \right) + 0.25 \cdot T \left( {{\text{C}}^\circ } \right) + 0.16 \cdot {\text{N}}_{2} {\text{-fixers}} \left( {{\text{mgC}} \cdot {\text{mL}}^{ - 1} } \right)$$

**Table 1:** VIP values of each variable.

	NO_3_	PO_4_	SiO_2_	Temperature	Salinity	Biomass	N_2_-fixers	Strat. index
PE-SYN	1.18	0.58	0.74	1.71	0.27	0.80	1.28	0.59
PC-SYN	1.54	0.51	0.27	1.47	0.81	0.15	0.44	1.49

The model for PC-SYN explained 40% of the cell abundance seasonal changes with 46% variation of the independent variables. The regression equation between PC-SYN cell abundance and the independent variables was (2):

2 $$y = - 0.28 \cdot {\text{NO}}_{3} \left( {\mu {\text{M}}} \right) + 0.13 \cdot {\text{T}} \left( {{\text{C}}^\circ } \right) + 0.35 \cdot {\text{N}}^{2} (10^{ - 3} {\text{s}}^{ - 2} )$$

### Picocyanobacteria community composition

**Figure 3:**
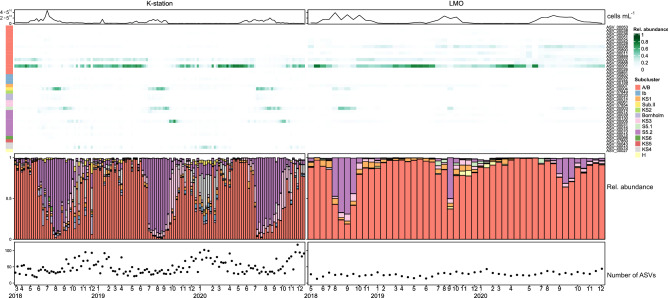
Relative abundance of ASVs with contribution > 1% to the total *Synechococcus* community in the K-station and LMO. The line plot on top represents total picocyanobacterial abundance for each sampling point. The heatmap represents the relative abundance of each ASV for each sampling point. The color code on the left indicates the clade of each ASV represented on the heatmap. The bottom barplot indicates the relative abundance of each subgroup/clade to the total *Synechococcus* community. The bottom dot plot indicates the number of ASVs for each sampling point.

In total, there were 2169 ASVs identified as SYN in the amplicon libraries. At the LMO, the number of ASVs remained more stable throughout the seasons (Fig. 3). The ASVs were affiliated with 13 different subgroups and clades: Bornholm, subalpine II, S5.1, S5.2, Ib and the newly defined A/B, KS1, KS2, KS3, KS4, KS5 and KS6. Clade A/B was defined as a combination between clade A and clade B, since the V5-V7 region of the 16S rRNA gene targeted in this study did not allow for a clear separation between these two clades. 39 ASVs represented > 1% of picocyanobacterial sequences in at least one sample, of which only one ASV belonged to the marine S5.1 clade (Supplementary Fig. S3), eight belonged to the S5.2 clade, 15 belonged to clade A/B and the rest were divided among freshwater clades (H, Subalpine I, Bornholm, Ib, KS1, KS2, KS3, KS4, KS5 and KS6). ASVs within clade A/B dominated at both stations during autumn (maximum contribution at the K-station 2018-11-10: 92% and LMO 2020-11-26: 92%), winter (maximum contribution at the K-station 2020-11-10: 90% and LMO 2019-02-19: 96%) and spring (maximum contribution at the K-station 2019-05-14: 98% and LMO 2020-05-26: 98%). During summer, the K-station libraries were dominated by S5.2 (maximum contribution 2019-08-13: 91%), followed by an increase in KS3 at the end of the summer to beginning of autumn (maximum contribution 2019-10-08: 68%). At the LMO the period of late summer to early autumn was occasionally dominated by S5.2 (maximum contribution 2018-09-11: 68%). The picocyanobacterial community was more diverse at the coastal K-station compared to the offshore LMO (K-station registered 1026 ASVs and LMO registered 195 ASVs). The phylogenetic classification (which only includes ASVs that represented > 1% of the sequences for at least one sampling point) showed that some sequences that belong to the clades KS2 (ASV00070), KS5 (ASV07850) KS6 (ASV00066) and S5.2 (ASV07854 and ASV07849) were only present at the coastal K-station. The number of ASVs was consistently higher at the K-station, with the highest numbers of ASVs present during autumn to early spring (Fig. 3).

### Correlations between environmental and biological variables with picocyanobacterial composition

**Figure 4:**
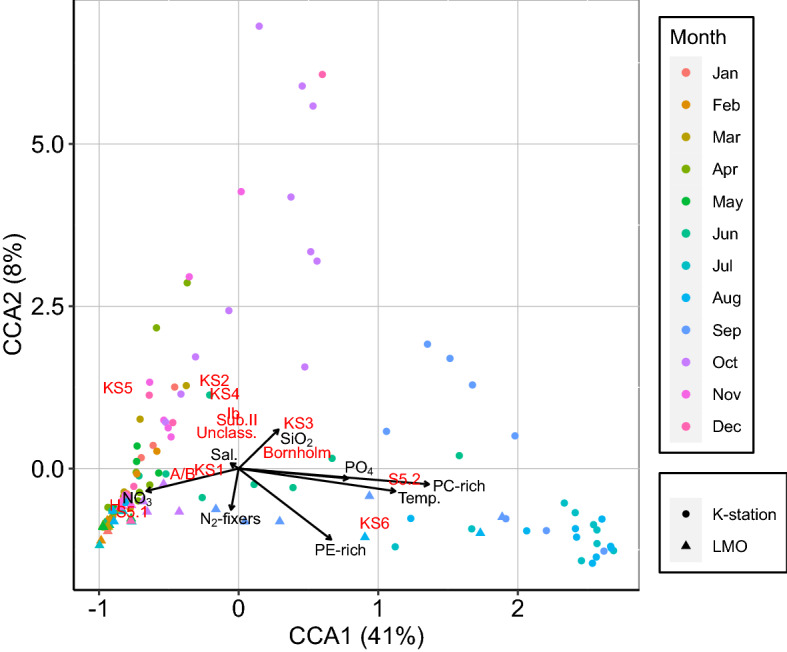
CCA analysis showing the relationships between environmental variables and picocyanobacterial community composition. Only variables that are significant are shown.

The test on Procrustes analysis and the CAs revealed a significant association between the distribution of the phylogenetic clades and the ASVs (squared m12 *P* value < 0.001, Supplementary Fig. S4). The stepwise CCA showed a significant relationship between clade composition and eight environmental and biological variables: NO_3_ (µM), SiO_2_ (µM), PO_4_ (µM), temperature (°C), salinity (PSU), N_2_-fixers biomass (mg C mL^−1^), PE-SYN (cells mL^−1^) and PC-SYN (cells mL^−1^; Table 2). The first two axis explained 49% of the taxonomic composition (Fig. 4). The first axis indicated a clear separation of summer samples. Summer samples were associated with high abundance of PE-SYN and PC-SYN, increase of temperature and high PO_4_. Autumn, winter and spring sites from the model clustered together and were associated with high NO_3_. PC-SYN abundance and temperature were the most important variables as indicated by the length of the variable arrows (Fig. 4).

**Table 2:** *P* values included in the CCA model after stepwise analysis.

	R2.adj	Df	AIC	F	*P* value
PC-SYN	**0.22**	**1**	** − 10.93**	**39.90**	**0.001**
Temperature	**0.28**	**1**	** − 20.21**	**11.50**	**0.001**
PO_4_	**0.33**	**1**	** − 29.96**	**11.91**	**0.001**
PE-SYN	**0.37**	**1**	** − 36.20**	**8.18**	**0.001**
N_2_-fixers	**0.38**	**1**	** − 38.86**	**4.53**	**0.003**
SiO_2_	**0.40**	**1**	** − 41.95**	**4.91**	**0.002**
NO_3_	**0.41**	**1**	** − 44.12**	**3.99**	**0.004**
PPE	0.43	1	− 45.15	2.86	0.018
N^2^	0.43	1	− 48.53	2.96	0.017
Salinity	**0.45**	**1**	** − 29.959**	**3.94**	**0.007**

Significant variables after the Holm correction are indicated in bold.

## Discussion

This study shows that temperature was one of the main parameters driving picocyanobacterial abundance. The correlation between temperature and increase in picocyanobacterial abundance is well known in marine^8,9^, estuarine^63,64^ and freshwater systems^65,66^. Picocyanobacterial abundance started increasing at > 10 °C, in line with other temperate ecosystems (e.g.^67^) and previous records in the Baltic Sea^9^. Peak abundances at the coastal K-station and offshore LMO during 2019 and 2020 were in the same range, with the exception of the summer of 2018, when K-station peak abundances reached 4.7 × 10^5^ cells mL^−1^. These numbers are comparable to other observations in the Baltic Proper during summer (10^5^ cells mL^−1^), and confirm previous observations that picocyanobacterial abundances are as high at coastal and offshore locations^55,57,68^. It is also important to note that the picocyanobacterial abundances at < 10 °C reported in this study were notably higher than previous reports in the Gulf of Finland and in other temperate ecosystems^8,9^. The thermal bound for SYN has recently been defined as > 5 °C^18^. However, in this study abundances of > 10^4^ cells mL^−1^ were recorded during winter time at both K-station (0–5.7 °C) and LMO (2.7–5.8 °C), suggesting that the strains present in the Baltic Sea are well adapted to low temperatures, in line with previous observations in marine^69^ and freshwater environments^70^.

According to the PLS models independent variables only explained 38 and 40% of the total variation of PE-SYN and PC-SYN respectively. This indicates that other important controllers such as light quality^4^, grazing by ciliates and flagellates^71^ or viral lysis^72^ may be important to include in future models. SYN was divided into PE-SYN and PC-SYN depending of the pigment content. PE-SYN and PC-SYN coexisted at similar abundances during the summer, confirming previous observations based on *cpcBA* and *cpeBA* libraries^29^. PE-SYN are better adapted to blue light which can penetrate deeper in the mixing layer, while PC-SYN are adapted to red light, which is dissipated at the surface ^4^. As a result, PE-SYN showed no correlation with the stratification index (N^2^) and was equally prevalent at both the K-station and the LMO. On the other hand, PC-SYN abundance variation was strongly linked to N^2^ and was more prevalent at the coastal K-station compared to the offshore LMO, in line with observations in other estuaries and freshwater lakes^11,73^. These results suggest a horizontal gradient in the Baltic Sea, where PC-SYN is more prevalent in coastal shallow areas while in offshore areas abundances are lower and tightly joined to stratification. In the future, an increase in the stratification periods as a result of global warming could reinforce PC-SYN dominance on the picocyanobacterial community. PC-SYN have been observed to avoid predation, viral lysis and to have a negative effect on co-occurring filter-feeders, which can affect the energy flow to upper trophic layers^74–76^. Thus, understanding the physiology and ecology of PC-SYN, a generally understudied group of SYN, is of high importance for the understanding of current and future climate scenarios.

Nutrient availability, particularly nitrogen species, was correlated to picocyanobacterial dynamics according to the PLS models. Both PE-SYN and PC-SYN showed moderate negative correlation with NO_3_ concentration. Some studies have suggested preference of picocyanobacteria for NH_4_ over NO_3_ at high temperature conditions (> 15 °C)^55,77^. Thus, newly fixed nitrogen in the form of NH_4_ from N_2_-fixers or regeneration during blooms may be an important driver supporting picocyanobacterial growth during summer^31–33^, in line with the positive correlation between N_2_-fixers and PE-SYN. However, PE-SYN peak abundances at the K-station and LMO were in the same range, although N_2_-fixers were only observed at the K-station during 2018. This suggests that PE-SYN can benefit from newly fixed nitrogen, but the presence of N_2_-fixers is not a requirement to achieve peak abundances and thus other nitrogen pools should also be considered. The main nitrogen source for picocyanobacteria during the summer may be regeneration through ammonification^34^. This may be especially important in coastal and shallow water areas (< 50 m depth) where studies have estimated that it can represent up to 97% of the nitrogen requirements^33,78^. In addition, peak abundances at the LMO were sustained during the first half of the autumn at < 10 °C, which indicates that picocyanobacteria can uptake NO_3_ at low concentrations efficiently^52^. Direct uptake of DON may also benefit SYN over other phytoplankton^35–37^.

The community composition was studied using 16S rRNA gene sequences amplified using specific primers that target almost exclusively picocyanobacteria^62^. The results corroborate that the V5-V7 region of the 16S rRNA gene showcases higher variability in picocyanobacteria than the V3-V4 region, revealing an unprecendented high strain diversity in the Baltic Sea with a particularly high number of ASVs at the coastal K-station. Most of the previously defined clades and clusters were described^62^, but some clades were not well resolved. For example, clades A and B clustered together (clade A/B) contrasting with phylogeny based on other regions of the 16S rRNA gene^12,79^. All ASVs in clade A/B displayed similar seasonal variation in relative abundance, suggesting a similar ecophysiology whithin the clade. The increase in contribution of S5.2 took place in June-July, when temperature was > 18 °C and NO_3_ concentration was low. This result suggests that S5.2 affiliated picocyanobacterial strains are adapted to high temperatures and may use NH_4_ as a primary nitrogen source^33,55,77^. On the other hand, clade A/B dominated during the colder months, indicating that picocyanobacteria strains in this clade have a preference for low temperatures and high NO_3_ concentration.

This study indicates a coastal to offshore differentiation in picocyanobacterial community composition. The coastal K-station presented higher ASV diversity than the offshore LMO. Moreover, the clades KS2, KS5 and KS6 were only present at the coastal K-station, which suggest that some clades are only present in the coastal region. These results contradict previous observations in the Baltic Proper where no significant differences in community composition were observed in coastal offshore gradients^6^. One explanation is that the higher resolution achieved with the primers in this study have revealed differences that could not be detected with less specific primers. Community composition seasonal dynamics in the coastal and offshore stations also showed major differences. At the K-station picocyanobacterial peak abundances took place when S5.2 was dominating the community while at the LMO, peak abundances took place under clade A/B dominance. The composition at the coastal compared to the offshore station could be driven by low PO_4_ levels as S5.2 was positively linked to PO_4_ concentration. At the LMO, PO_4_ was low during summer (mean summer concentration: 0.17 µM) while it remained high at the K-station (mean summer concentration: 0.63 µM), explaining the lower contribution of S5.2 in the LMO. The potential PO_4_ limitation could also explain why at the LMO S5.2 has lower contributions during summer to the community composition compared to the K-station. However, to fully understand picocyanobacterial dynamics, other parameters such as light hours^80^, NH_4_ recycling rates^33^ or nutrient competition with specific phytoplankton groups^77^ should also be considered.

The most abundant ASV in the dataset, ASV_00001, was identical to the V5-V7 region of a metagenome-assembled genome (MAG) reconstructed from the Baltic Proper (BACL30) and has been identified as dominant in the Baltic Sea^16,60^. Phylogenetic classification based on six ribosomal proteins included BACL30 in the S5.2 clade^16^; however this classification may have been biased by the lack of genome sequenced freshwater SYN strains. In this study, ASV_00001 had 99% identity with the strain MW73D5, a freshwater strain included in clade A/B^12^. Most of the ASVs of the picocyanobacterial community in the Baltic Sea were more similar to freshwater strains rather than estuarine and marine strains. Hugerth et al.^60^ studied bacterial MAG phylogeny (including BACL30 as the only representative for SYN) from brackish environments and observed that a significant proportion of the MAGs had closest affiliation to strains of brackish origin. The affiliation of ASV_00001 to clade A/B (freshwater) suggests that Baltic Sea picocyanobacteria have evolved from freshwater strains. ASV_00001 showed high contributions during the summer, particularly at the LMO (maximum relative contribution: 78%), in line with observations in other offshore locations^15,16^. However, the highest contributions took place in the cold months, indicating that BACL30 is well adapted to low temperature conditions.

This study provides a detailed description of picocyanobacterial seasonal abundance and community composition during three years at a coastal and an offshore station in the Baltic Proper, showing that SYN are highly adaptable and diverse. The results link SYN populations to environmental parameters. The Baltic Sea is warming up faster than other oceans^81^, and it is predicted that metereological summer in the Baltic Proper will take place 20 days earlier by 2100^82^. Longer and warmer summers could result in earlier picocyanobacterial blooms in the coast as a consequence of achieving optimal temperatures for S5.2 ecotypes earlier in the spring/summer season. This effect could be further magnified by earlier and more extensive blooms of N_2_-fixing cyanobacteria resulting in higher NH_4_ availability, which are projected as a consequence of global warming^82,83^. However, at offshore locations in the Baltic Proper, the picocyanobacterial summer bloom could be delayed since optimal temperature for clade A/B would take place later in the year. In future climate conditions, the contribution of picocyanobacteria to the carbon pools is expected to increase while bigger phytoplankton (PPE and other protists) are expected to decrease^84,85^. These changes in community composition together with potential changes in SYN peak abundances timing could result in significant alterations of picophytoplankton grazers, and consequently the rest of the trophic chain^86^. The results in this study highlight that besides temperature, water column stratification and nutrient availability also play an important role in picocyanobacterial dynamics and community composition.

## Methods.

### Field sampling

Water was sampled every second week at the Linnaeus Microbial Observatory (LMO, 56° 55′ 51.24″ N, 17° 3′ 38.52″ E, 2 m sampling depth), an offshore station located 10 km off the east coast of Öland (Sweden) and weekly at the K-station, a coastal station located in the city of Kalmar, Sweden (56° 39′ 25.4″ N 16° 21′ 36.6″ E, 1 m sampling depth) during 2018–2020. The temperature and salinity were measured using a conductivity/temperature/depth sensor CTD® Castaway at the K-station and a CTD probe (AAQ 1186-H, Alec Electronics, Japan) at the LMO. To remove large particles, samples were filtered through a 200 µm mesh.

### Nutrients and Chl a

Water for measuring dissolved inorganic nutrients (NH_4_, NO_2_ + NO_3_ (referred to as NO_3_), PO_4_ and SiO_2_,) was sampled and frozen at − 20 °C until analysis using standard protocols (UV-Spectrophotometer^87^). For measuring Chl *a*, 50–200 mL seawater was filtered through duplicate 25 mm A/E glass fiber filters (∼1 μm pore size, Pall life Sciences, Ann Arbor, MI, USA). Filters were incubated overnight in darkness in 5 mL of ethanol (96%) and fluorescence was measured the following day using a fluorometer (Turner design Model #040, Tucson, USA) following the Jespersen & Christoffersen^88^ protocol.

### Phytoplankton abundance and community composition

Samples for phytoplankton identification (> 5 µm diameter) were fixed in acid lugol (1% final concentration) and counted according to the Utermöhl method^89^ using an inverted microscope (Nikon TMS, Tokyo, Japan). The phytoplankton carbon biomass concentration (mg C mL^−1^) was derived from the cell abundance and carbon biomass^89,90^. In addition to the traditional taxonomy the genus *Aphanizomenon*, *Nodularia* and *Dolichospermum* were included in the group defined as N_2_-fixers.

### Picophytoplankton abundance

Samples to determine picophytoplankton abundance were fixed with glutaraldehyde solution Grade I 25% in H2O (Sigma-Aldrich, Missouri, USA; 1% final concentration) and stored at − 80 °C until analysis. K-station samples until 12th May 2020 and LMO samples until 10th July 2019 were analyzed using a Cyflow® Cube8 flow cytometer (Partec®, Germany) at 10 µL s^−1^ while after that date a BD FACSverse (BD Biosciences) was used instead. Picophytoplankton were counted as three populations: photosynthetic picoeukaryotes (PPE), phycoerythrin rich (PE-SYN) SYN cells and phycocyanin rich (PC-SYN) SYN based on the gating described in Alegria Zufia et al.^55^. The observations for PPE abundance variation are presented in Supplementary Figure S1. Gating and visualization of the flow cytometric data were carried out using FCSalyzer ver. 0.9.22-alpha^91^.

### DNA extraction, PCR amplification and sequencing

Samples for DNA analysis were collected by filtering 400 mL of water through a 0.2 µm Supor®-200 filter (Pall Corporation, USA). The filters were stored at − 80 °C until extraction. DNA was extracted using the FastDNA™ SPIN Kit for Soil from MP Biomedicals Inc. according to manufacturer´s instructions with the addition of an incubation with proteinase-K (0.02 µg/µL, final concentration) at 55 °C for one hour. Sample concentration was measured using an Invitrogen Qubit 2.0 fluorometer (Thermo Fisher Scientific Inc.). Sample purity was assessed using a Thermo Scientific™ NanoDrop 2000 spectrophotometer (Thermo Fisher Scientific Inc.). The V5-V7 hypervariable region of the 16 rRNA gene was amplified using the primers Cya-771F (5’-AGGGGAGCGAAAGGGATTA-3’) and Cya-1294R (5’-GCCTACGATCTGAACTGAGC-3’) described in^62^. The PCR reaction was prepared in duplicates for each sample using the Thermo Scientific Phusion High-Fidelity PCR Master Mix according to the manufacturer’s instructions with a reaction volume of 25 µL. The PCR was performed on a T100™ Thermal Cycler (BIO RAD, USA) with an initial denaturation at 98 °C for 30 s; 20 cycles of denaturation at 98 °C for 10 s, annealing at 55 °C for 1 min and extension at 72 °C for 5 s; and a final extension step at 72 °C for 2 min. Amplicon sequences were purified with AMPure XP (Beckman Coulter, USA) according to manufacturer’s instructions prior to performing the index PCR. Indexes were attached to individual samples using NEXTERA Dual indexes (Illumina Inc.) in a PCR with an initial denaturation at 98 °C for 30 s; 12 cycles of denaturation at 98 °C for 10 s, annealing at 62 °C for 30 s and extension at 72 °C for 5 s; and a final extension step at 72 °C for 2 min. Amplicon sequences were purified again after index PCR with AMPure XP (Beckman Coulter, USA). The purified amplicons were quantified with Invitrogen Qubit 2.0 fluorometer (Thermo Fisher Scientific Inc.) and pooled at equimolar concentrations. Indexed samples were sequenced with Illumina MiSeq (Illumina Inc, USA) with 300 cycle paired-end sequencing.

### Bioinformatics processing

The resulting reads were denoised and screened for chimera removal with ampliseq (v1.1, https://github.com/nf-core/ampliseq) which runs on QIIME2 (2019.10)^92^ and DADA2 (1.10.0)^93^ and taxonomy assignment of the resulting amplicon sequencing variants (ASVs) were done using the SILVA 132 database with a 90% identity threshold. Among all sequences in the libraries, 95% belonged to cyanobacteria, of which 97% were classified as Synechococcales. Then, a phylogenetic tree was constructed with the aligned sequences to cross check that all of them indeed affiliated with Synechococcales; sequences that did not affiliate were excluded from further analyses. The number of sequences for each sample after each step of the quality control pipeline are specified in Supplementary Table S1.

### Phylogenetic analysis

The ASVs that represented > 1% of the sequences in at least one sampling point were selected. To determine the phylogenetic clade affiliation, the closest representative sequences were identified and retrieved using the BLASTn-Search engine in the NCBI database. Sequences were aligned using MAFFT v.7^94^ and a phylogenetic tree was constructed by the maximum likelihood method (ML) in MEGAX software^95^ following the GTR + G + I model (bootstrap values inferred from 1000 replicates). ASVs were associated to a specific clade or subgroup when they were located in a (mostly) monophyletic branch that contains a well defined reference sequence associated to that clade. References for clades were obtained from Huber et al.^62^, Crosbie et al.^96^, Silva et al.^97^, Marsan et al.^98^ and Choi et al.^45^. Branches that did not associate with previously known clades were given new clade names. The resulting tree was edited using the interactive tree of Life (iTOL, http://itol.embl.de).

### Statistical analysis

Datasets were rarefied to a sequence depth of 27,819 sequences per sample. All statistical analyses were performed using R version 3.6.1^99^ and the vegan package^100^. All plots were produced using the ggplot2 (3.3.6)^101^, and ComplexHeatmap (3.15)^102^.

#### Partial least square regression

Partial least square regression (PLS) was used to evaluate the picocyanobacterial relationship with the following independent variables: in situ nutrients (NO_3_ (µM), PO_4_ (µM), SiO_2_ (µM)), temperature (°C), salinity (PSU), stratification index (N^2^; 10^–3^ s^−2^), presence of N_2_-fixers (mg C mL^−1^) and total phytoplankton biomass (mg C mL^-1^). The model was performed separately for PE-SYN (cells mL^−1^) and PC-SYN (cells mL^−1^) separately and by pooling together the data from K-station and LMO. All variables were log_10_(x + 1) transformed for standardization. The stratification index was calculated following the Brunt-Väisälä frequency (3)^103^:

3 $$N^{2} = - \frac{g}{\rho }\frac{{\rho_{s} - \rho_{b} }}{H}$$

where *g* is the gravitational acceleration (m s^−2^), *ρ* is the average density of seawater (1.025 g cm^−3^), *ρ*_*s*_ is the surface density (g cm^−3^) and *ρ*_*b*_ is the density at the bottom (g cm^−3^). The K-station is 3 m deep, and thus all the water column is permanently in the photic zone. However, the calculated N^2^ was ~ 0, indicating an unstable water column. Because of this, 1 × 10^–3^ s^−2^ values were assigned to all sampling dates in the K-station, indicating a strong stratification. The variable importance in projection (VIP) was used to determine the relative importance of the independent variables considered^104,105^. Variables were considered significant when VIP > 1.

#### Procrustes analysis

Procrustes analysis was used to compare correspondence analysis (CA) based on phylogenetic clades and ASVs. The goal of this analysis was to test whether the distribution of the phylogenetic clades was congruent with the distribution of ASVs. The significance of the association of the two datasets was later explored with a squared m12 test (999 permutations, *P* value < 0.05).

#### Canonical correspondence analysis

A step-wise canonical correspondence analysis (CCA) (*P* value = 0.05) was performed to identify the relationship of environmental and biotic variables to SYN community clade composition. The variables considered were: NO_3_ (µM), PO_4_ (µM), SiO_2_ (µM), temperature (°C), salinity (PSU), stratification index (10^–3^ s^−2^), phytoplankton biomass (mg C mL^−1^), N_2_- fixers biomass (mg C mL^−1^), PE-SYN (cells mL^−1^) and PC-SYN (cells mL^−1^). To avoid type I errors, results were considered significant when *P* value after Holm correction resulted in *P* value < 0.05. All the independent variables were log_10_(x + 1) transformed for standardization.

## Supplementary Information

Supplementary Information.

## Data Availability

The datasets generated and/or analysed during the current study are available in the NCBI repository, BioProject PRJNA810944.
