# Supplementary material for: Seasonal dynamics in picocyanobacterial abundance and clade composition at coastal and offshore stations in the Baltic Sea
Source: Sci Rep. 2022 Aug 22;12:14330. doi: 10.1038/s41598-022-18454-8 (PMC9395346; doi:10.1038/s41598-022-18454-8)
Supplement: Supplementary file 1 — Supplementary Information. [file 41598_2022_18454_MOESM1_ESM.pdf]

Seasonal dynamics in picocyanobacterial abundance and clade composition at coastal and offshore stations in the Baltic Sea

Javier Alegria Zufia[1], Catherine Legrand[1,2] & Hanna Farnelid[1*]

[1]*Department of Biology and Environmental Science, Centre for Ecology and Evolution in Microbial Model Systems (EEMiS), Linnaeus University, Kalmar, Sweden*

[2]*School of Business, Innovation and Sustainability, Halmstad University, Halmstad, Sweden*

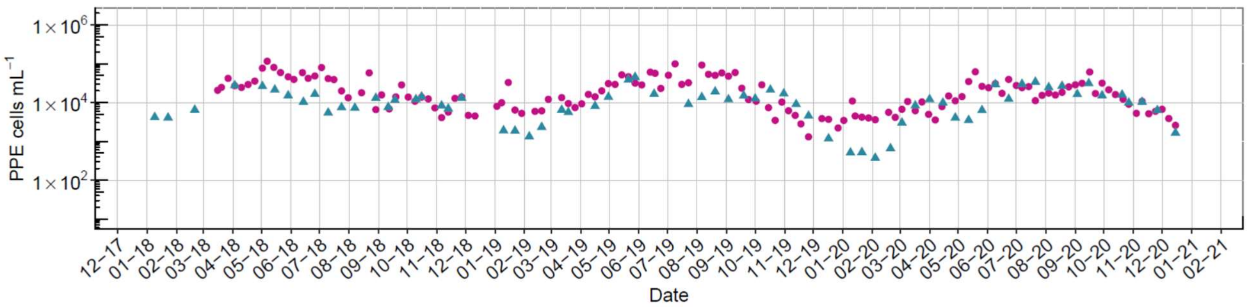

Supplementary Fig. S1. K-station and LMO measurements for PPE (cells mL^{-1})

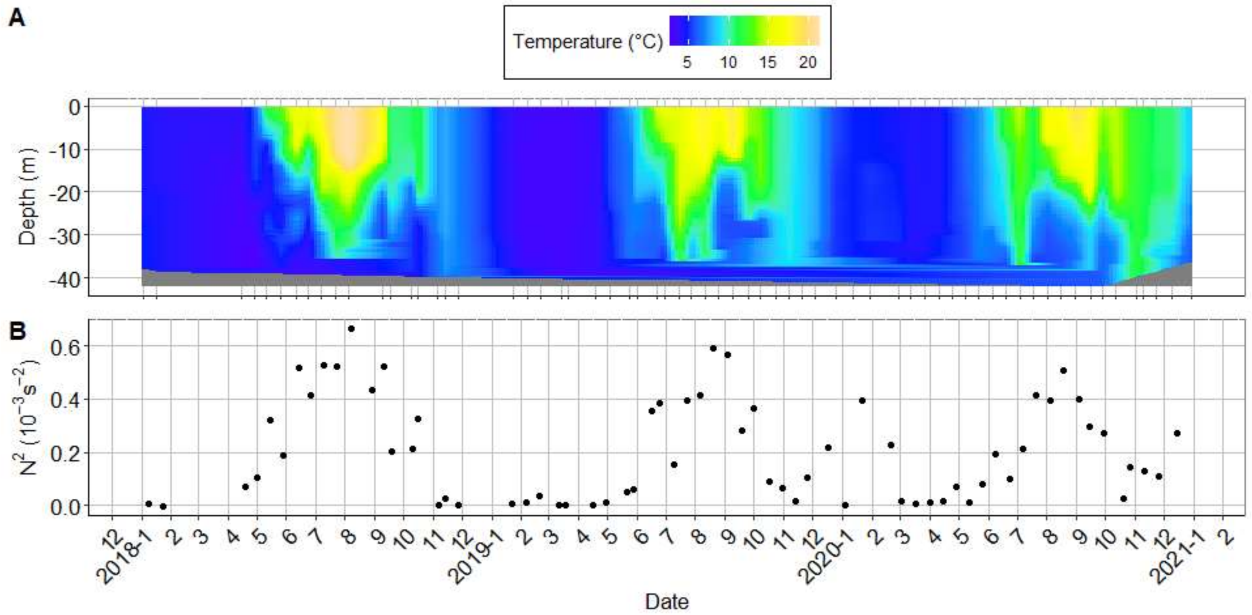

Supplementary Fig. S2. K-station and LMO measurements for (A) vertical profile temperature in the LMO

and (B) stratification index (N^2).

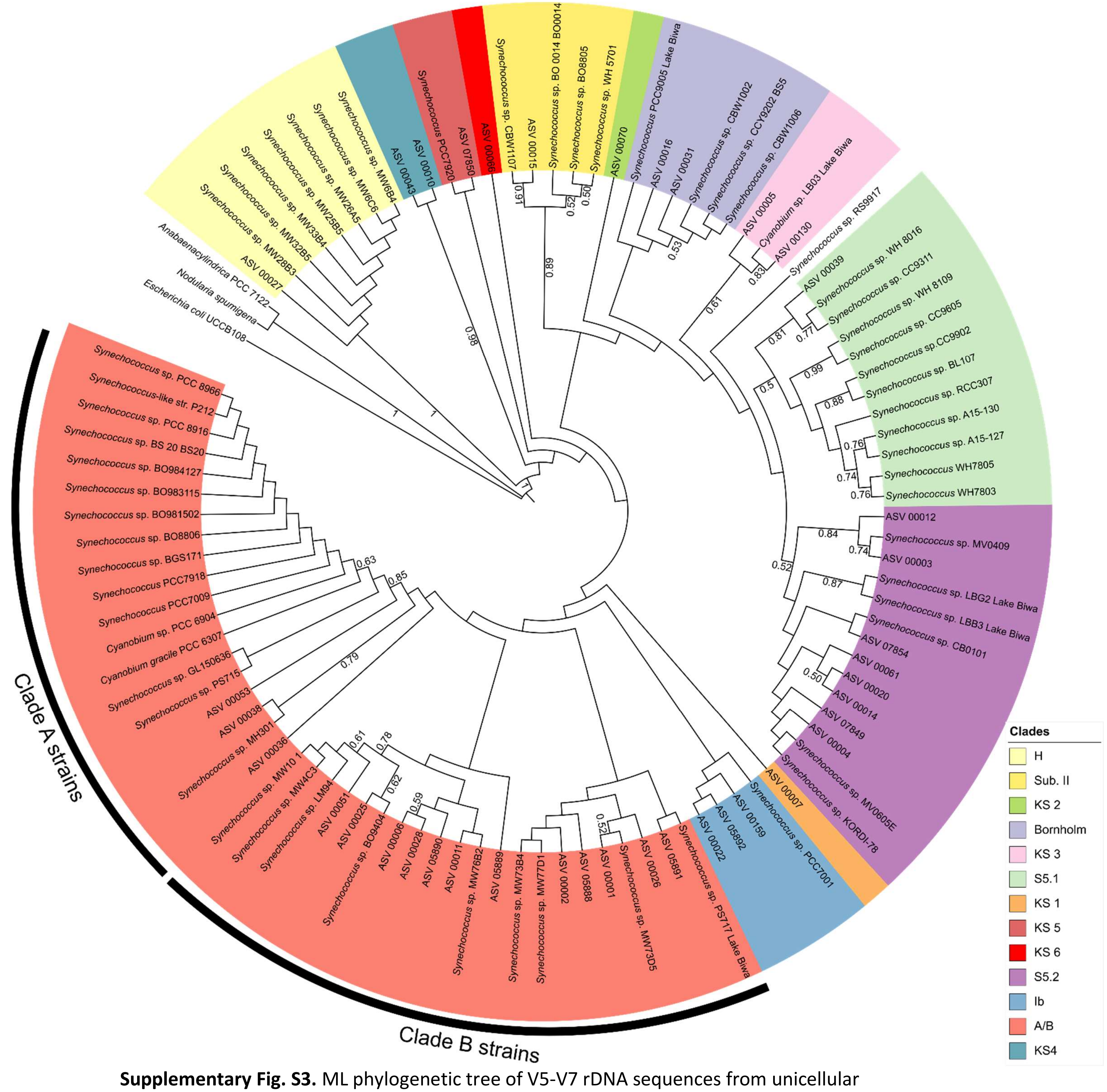

Supplementary Fig. S3. ML phylogenetic tree of V5-V7 rDNA sequences from unicellular picocyanobacteria.

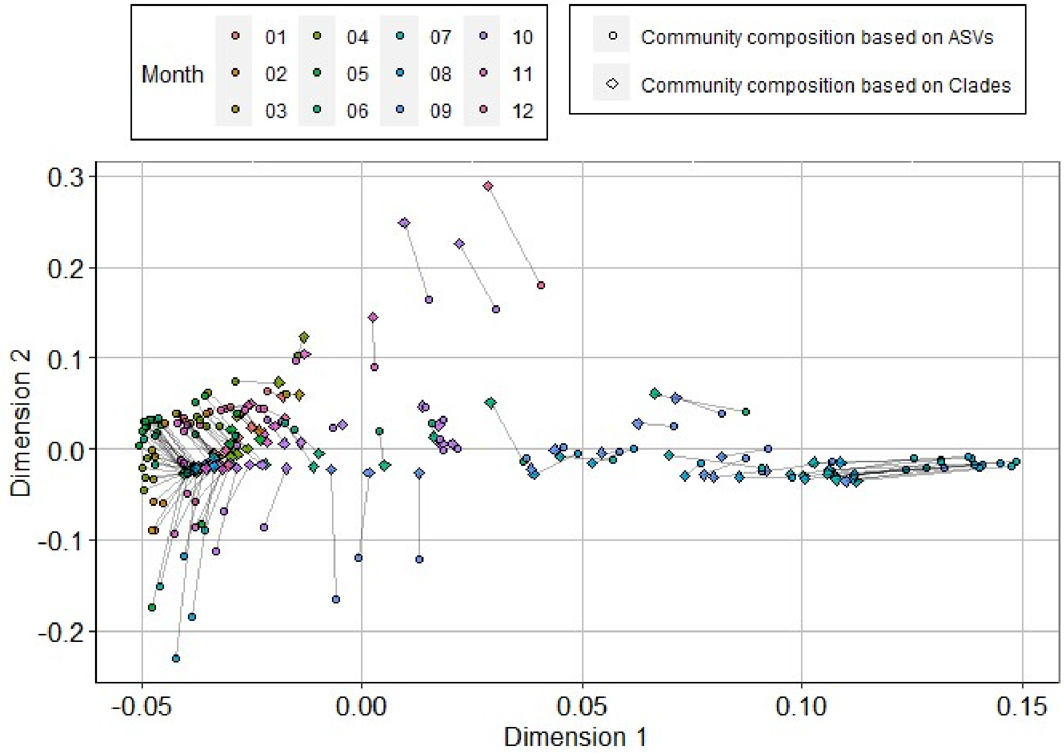

Supplementary Fig. S4. Graphical representation of the first two dimensions of the Procrustes analysis between CA based on ASV community composition and CA based on clade community composition.

Supplementary Table S1. Number of sequences from the V5-V7 amplification for each sample after each step of the quality control pipeline.

Sample-ID	Raw sequences	Filtered sequences	Denoised sequences	Merged sequences	Non-chimeric sequences
KS1	218008	159268	158590	156090	93953
KS10	132928	102630	102468	102174	69157
KS100	197267	146646	144382	138995	94787
KS101	172995	146981	146013	143902	100776
KS102	207630	164134	161230	155668	81872
KS103	168871	127945	126254	122434	87662
KS104	204153	159743	157730	153518	80549
KS105	165123	140088	139136	137392	98486
KS106	205277	171221	169904	167157	108671
KS107	155590	127615	126275	123256	78814
KS108	135450	107258	106224	104256	73433
KS109	116790	96670	96228	95615	77034
KS11	131865	101723	101548	101433	71777
KS110	257310	175823	173174	166445	114964
KS111	249021	181199	180072	176527	141225
KS112	244010	180465	180120	179257	164713
KS113	219798	150888	150356	148476	129457
KS114	241724	168807	168537	168035	152438
KS115	245376	177395	176687	175061	144912
KS116	169123	119885	119677	119329	101434
KS117	170926	125568	125393	125145	105891
KS118	226262	158974	158737	158393	130353
KS119	244264	179311	178615	177499	98856
KS120	215164	156941	156616	156043	101916

KS121	224106	153310	152987	152622	97076
KS122	234836	161980	161155	160217	78946
KS123	283668	203574	202603	200558	132833
KS124	182286	128908	128703	128466	89642
KS125	199834	144377	144122	143739	96440
KS126	225411	173438	172731	171733	80654
KS127	300470	238744	238148	237317	112167
KS128	252407	199124	198623	198212	100271
KS129	185886	140206	139901	139629	61665
KS13	120309	86718	86587	86304	61926
KS130	269063	203738	203332	202958	119390
KS131	273080	213129	212375	212113	117054
KS132	275475	203894	201422	197373	95560
KS133	253198	195328	194647	194058	104798
KS134	199117	151214	150293	148747	69957
KS136	178007	138763	138007	136852	58316
KS137	171977	119060	118501	117849	55034
KS138	165463	122900	121594	119362	47924
KS139	168185	121939	119779	114642	51364
KS14	123234	88456	88191	87951	56685
KS140	217468	150196	147939	143132	73438
KS141	194600	147566	146983	146191	92639
KS142	293816	209184	205892	196797	109063
KS143	314357	234091	229589	220087	105254
KS144	253228	193518	190892	185952	90727
KS145	228529	165484	162916	157819	76395
KS146	263193	192197	188970	182892	91017
KS16	136621	100112	99774	99198	58631

KS18	101246	73703	73451	73248	34180
KS19	125902	92737	91764	91247	44000
KS2	199879	148726	148123	146575	95071
KS20	202199	153986	153131	152698	55362
KS21	218940	173713	173020	172791	96195
KS22	216002	158876	158652	158476	147810
KS23	185975	135022	134640	134453	86540
KS24	187988	140140	139648	139349	71832
KS25	186196	141380	140997	140792	87106
KS26	176886	133764	133604	133526	88629
KS27	183349	140574	139927	139759	83827
KS28	197695	142784	142448	142243	109350
KS29	213640	162699	162110	161673	82308
KS3	186757	140003	139404	138093	84356
KS30	180861	136763	136459	136197	74346
KS31	251911	173461	172121	170211	78209
KS32	228467	161405	160707	159561	81748
KS33	238687	173368	172986	172226	130138
KS34	209337	148354	147920	147056	107471
KS35	195705	131269	129633	125632	70183
KS36	243027	183348	182981	181794	106179
KS37	250083	185500	183892	179611	82761
KS38	294410	226878	226159	224350	128513
KS39	227419	160479	159441	157138	88873
KS4	176252	124012	123652	122669	85878
KS40	269455	185448	183614	178150	89810
KS41	330461	244242	243059	240071	134187
KS42	273718	201597	201178	199929	132878

KS43	268832	189918	187687	182940	95827
KS44	242904	182132	181764	181000	105734
KS45	243809	188708	188198	187096	118608
KS46	271983	206633	205804	203994	119178
KS47	224871	159060	159002	158132	130612
KS48	257800	188448	187667	186712	107576
KS49	274200	197717	195624	192314	79578
KS5	234576	164501	163687	161611	105109
KS50	267010	192083	190758	188496	91026
KS51	265340	192966	191544	188527	102380
KS52	144776	105997	105378	104295	56165
KS53	160520	123560	123175	122612	76071
KS54	159734	117333	116343	114336	54181
KS55	139511	93238	92761	91801	74966
KS56	159664	111498	111221	110734	92448
KS57	146916	98143	97343	95426	71169
KS58	177976	121501	120205	116831	57996
KS59	151007	111427	111113	110648	78233
KS6	230647	167498	166600	164728	98660
KS60	216945	158168	157713	156637	97035
KS61	209757	160133	159861	159349	111461
KS62	212096	161320	161031	160514	110859
KS63	187970	121588	120943	118930	90407
KS64	196437	141425	141171	140831	106881
KS65	188066	138140	137916	137657	103490
KS66	184091	127870	127258	126022	99975
KS67	208659	152958	152614	151968	105675
KS68	151163	113032	112701	112486	80103

KS69	97305	74913	74582	74183	38178
KS7	162804	118653	118419	117438	71650
KS70	159726	123023	122487	121970	50689
KS71	154433	107930	107366	106742	70373
KS72	169620	117051	116772	116453	100677
KS73	139683	104052	103606	103471	70063
KS74	156610	113773	113406	112970	73758
KS75	147410	109063	108579	107953	62637
KS76	163771	118340	118145	117763	94822
KS77	170058	125872	125201	123810	82156
KS78	132505	88185	88148	88085	87479
KS79	200716	139956	139398	139040	88873
KS8	197585	141057	140266	137492	78133
KS80	161940	113337	113089	112913	95092
KS81	171360	120952	120572	119568	96440
KS82	166131	116140	115640	115176	86677
KS83	161100	112333	111414	109216	73674
KS84	206365	158131	157707	157042	112220
KS85	204786	159150	158646	157176	119108
KS86	238438	173576	172289	169087	123984
KS87	202504	149886	149497	148605	112347
KS88	242577	177912	177178	175778	126270
KS89	232938	165078	163800	159281	106804
KS9	110321	77086	76471	74834	44337
KS90	215602	161880	161204	160258	110679
KS91	205468	153961	153252	151361	105961
KS92	210974	139124	137026	131436	77082
KS93	203924	150211	148520	144797	89783

KS94	218901	152287	149801	144351	82294
KS95	190556	135754	135014	133910	85149
KS96	205303	140054	137868	133162	41999
KS97	213455	148397	146490	143102	72861
KS98	187876	127918	126220	122540	45729
KS99	223056	155110	152880	148448	66013
LMO-2018-04-03	111693	97708	97592	97370	65091
LMO-2018-04-18	109001	90978	90864	90503	66800
LMO-2018-05-02	117169	98272	98048	97446	59425
LMO-255	117851	101459	101227	100997	59723
LMO-257	57941	50458	50443	50338	29048
LMO-258	152114	132589	132276	132055	50255
LMO-260	137147	121534	121168	121004	55030
LMO-262	196108	174466	173473	173387	86622
LMO-263	153282	132322	132205	132148	81409
LMO-264	166588	144463	144430	144398	75182
LMO-265	155757	137109	137045	136937	77388
LMO-267	152186	133713	133610	133372	78120
LMO-268	138763	123007	122933	122838	67773
LMO-269	189114	161755	161639	161349	87916
LMO-271	219002	187571	187222	186660	128281
LMO-273	197016	170997	170919	170635	124410
LMO-274	162873	134377	134332	134227	111349
LMO-276	223388	188575	188394	187970	143523
LMO-277	138029	115692	115543	115392	94754
LMO-278	194720	164920	164776	164516	108051
LMO-279	157486	135059	134919	134598	76546
LMO-280	214101	184995	184829	184577	129791

LMO-281	159897	139471	139336	139013	76937
LMO-282	199057	172486	172192	171998	98758
LMO-284	161528	136376	136185	135948	69098
LMO-285	182263	155523	155251	155047	84690
LMO-288	182127	155305	155170	154834	75470
LMO-290	176800	153695	153580	153068	81895
LMO-292	181698	158144	157945	157145	83188
LMO-293	212539	182001	181710	180976	102532
LMO-294	207452	176820	176448	175746	125492
LMO-295	216371	181892	181803	181524	146670
LMO-296	178185	139746	139393	138995	118962
LMO-297	163141	124958	124891	124755	122152
LMO-298	210888	177541	177408	177057	141262
LMO-299	214675	182063	181843	181481	140796
LMO-300	182088	157934	157687	157402	106487
LMO-301	133743	110893	110730	110499	87657
LMO302	211334	162267	162083	161946	104820
LMO303	157268	110651	110342	109192	71301
LMO304	110450	83863	83611	82964	45342
LMO305	187271	142217	142005	141685	113089
LMO309	218301	160754	160175	159857	74318
LMO310	171220	130635	129951	129174	35015
LMO311	194434	145357	145044	144728	65336
LMO312	105105	81342	81202	81108	37554
LMO313	273842	212330	212179	211899	113521
LMO314	293882	234762	234397	234069	123006
LMO315	236617	188688	188435	188042	113687
LMO316	211962	160402	160201	159659	112027

LMO317	253475	193436	193036	192314	120191
LMO318	264014	201539	201218	200056	125130
LMO319	241298	183592	183062	181991	130538
